# Novel miR-b2122 regulates several ALS-related RNA-binding proteins

**DOI:** 10.1186/s13041-017-0326-7

**Published:** 2017-10-02

**Authors:** Zachary C. E. Hawley, Danae Campos-Melo, Michael J. Strong

**Affiliations:** 1 Molecular Medicine Group, Robarts Research Institute, Schulich School of Medicine and Dentistry, Western University, London, Ontario Canada; 2 Department of Clinical Neurological Sciences, Schulich School of Medicine and Dentistry, Western University, London, Ontario Canada; 3 Department of Pathology, Schulich School of Medicine and Dentistry, Western University, London, Ontario Canada; 4 Rm C7-120 LHSC, University Hospital, 339 Windermere Road, London, Ontario N6A 5A5 Canada

**Keywords:** Amyotrophic lateral sclerosis (ALS), Motor neuron, miRNAs, TDP-43, FUS/TLS, RGNEF, mRNA stability, MotomiRs, Neurodegeneration

## Abstract

Common pathological features of amyotrophic lateral sclerosis (ALS) include cytoplasmic aggregation of several RNA-binding proteins. Out of these RNA-binding proteins, TDP-43, FUS/TLS and RGNEF have been shown to co-aggregate with one another within motor neurons of sporadic ALS (sALS) patients, suggesting that there may be a common regulatory network disrupted. MiRNAs have been a recent focus in ALS research as they have been identified to be globally down-regulated in the spinal cord of ALS patients. The objective of this study was to identify if there are miRNA(s) dysregulated in sALS that are responsible for regulating the TDP-43, FUS/TLS and RGNEF network. In this study, we identify miR-194 and miR-b2122 to be significantly down-regulated in sALS patients, and were predicted to regulate *TARDBP*, *FUS/TLS* and *RGNEF* expression. Reporter gene assays and RT-qPCR revealed that miR-b2122 down-regulates the reporter gene through direct interactions with either the *TARDBP*, *FUS/TLS*, or *RGNEF* 3’UTR, while miR-194 down-regulates firefly expression when it contained either the *TARDBP* or *FUS/TLS* 3’UTR. Further, we showed that miR-b2122 regulates endogenous expression of all three of these genes in a neuronal-derived cell line. Also, an ALS-associated mutation in the *FUS/TLS* 3’UTR ablates the ability of miR-b2122 to regulate reporter gene linked to *FUS/TLS* 3’UTR, and sALS samples which showed a down-regulation in miR-b2122 also showed an increase in FUS/TLS protein expression. Overall, we have identified a novel miRNA that is down-regulated in sALS that appears to be a central regulator of disease-related RNA-binding proteins, and thus its dysregulation likely contributes to TDP-43, FUS/TLS and RGNEF pathogenesis in sALS.

**Electronic supplementary material:**

The online version of this article (doi: 10.1186/s13041-017-0326-7) contains supplementary material, which is available to authorized users.

## Background

Amyotrophic lateral sclerosis (ALS) is a progressive motor neurodegenerative disease resulting in paralysis and death within 2–5 years after diagnosis [1, 2]. 5–10% of ALS cases are familial (fALS), while the remaining are sporadic (sALS) although ~10–12% of these latter cases also have a genetic basis [1, 3, 4]. While our understanding of ALS pathogenesis has advanced significantly in recent years, this understanding, and in particular the relationship amongst the individual genetic defects and the associated formation of pathological intraneuronal inclusions, which are a hallmark of the disease, remains in its early phases [5–7].

Defects in mRNA metabolism has been suggested to be a major driver in the genesis of pathological inclusions within ALS [8–12]. Further, it has been shown that miRNAs, essential regulators of mRNA expression and protein synthesis, are globally down-regulated within the spinal cord tissue of sALS patients [13, 14]. This down-regulation of miRNA expression has been shown to be motor neuron specific [15], contributing to the concept that altered miRNA homeostasis is a major contributor to the pathogenesis of ALS [16, 17]. The finding of this global down-regulation of miRNAs within sALS patients is intriguing, as TDP-43 and FUS/TLS, two proteins often found to be dysregulated in sALS, are known to be essential components of miRNA biogenesis [18, 19]. Further, ALS mutations within the FUS/TLS 3′ untranslated region (UTR) have been shown to disrupt a negative feedback network between miR-141/200a and FUS/TLS, leading to accumulation of FUS/TLS within the cell [20, 21]. This suggests that there may be a disruption in the feedback networks between miRNAs and RNA-binding proteins in ALS, including TDP-43 and FUS/TLS.

Beyond TDP-43 and FUS/TLS, we have described RGNEF, another RNA-binding protein, that forms pathological aggregates within motor neurons of sALS spinal cord and has mutations associated with ALS [6, 22–24]. Interestingly, we observed that TDP-43, FUS/TLS and RGNEF co-aggregate with each other within the motor neurons of sALS patients, suggesting a co-dysregulation of these three RNA-binding proteins [6]. While miRNA biogenesis has been clearly shown to be affected in sALS, it is unclear the consequence of this mass down-regulation, and how it may contribute to TDP-43, FUS/TLS and RGNEF pathogenesis.

In the current study, we describe two miRNAs, miR-194 and miR-b2122, that are predicted to regulate TDP-43, FUS/TLS and RGNEF. The novel miR-b2122 is expressed in human spinal motor neurons, is significantly down-regulated in sALS patients, and regulates the expression of all three of these RNA-binding proteins. Further, an ALS-associated mutation within the *FUS/TLS* 3’UTR is located in the miRNA recognition element (MRE) of miR-b2122 and disrupts its ability to suppress gene expression. Overall, our results suggest that the down-regulation of miR-b2122 within sALS cases could result in altered levels of all three of these RNA-binding proteins, contributing to the pathological state of TDP-43, FUS and RGNEF observed within motor neurons of sALS patients.

## Methods

### Tissue samples

Spinal cord tissue was obtained from sALS patients and age-matched, neurologically intact individuals. All ALS cases were both clinically and neuropathologically confirmed using the El Escorial Criteria (World Federation of Neurology Research Group on Neuromuscular Disease, 1994). All research was approved by “The University of Western Ontario Research Ethics Board for Health Sciences Research Involving Human Subjects (HSREB)”. Written consent for autopsy was obtained from the next of kin at the time of death or from the patient antemortem in accordance with the London Health Sciences Centre consent for autopsy. Cases were genotyped and confirmed to have no known mutations in *SOD1*, *TARDBP*, *FUS/TLS*, *RGNEF* or expanded repeats in *C9orf72.*

### 3′ race

Total RNA extraction was performed on SH-SY5Y cells and spinal cord tissue from neurologically intact humans using TRIzol reagent (Life Technologies Inc., Ambion, Carlsbad, CA, USA). This was followed by cDNA synthesis and PCR with the SMARTer 5′/3′ RACE Kit (Takara Bio. Inc., Clontech, USA) to amplify the *TARDBP*, *FUS/TLS* and *RGNEF* 3’UTRs according to the manufactures instructions using the following forward primers: *TARDBP* 5′-TAG ACA GTG GGG TTG TGG TTG GTT GGT A-3′, *FUS/TLS* 5′- GCA GGG AGA GGC CGT ATT AAT TAG CCT-3′ and *RGNEF* 5′-GCC CCG AGG TAA TGG AAC TTA ATC G-3′. 3’UTRs were identified using a 1% agarose gel containing a SYBR Safe dye. 3’UTR bands were excised and extracted from the agarose gel, and then individually cloned into a pGEMT-easy vector according to manufactures instructions (Promega, Madison, WI, USA). All 3’UTRs were confirmed using Sanger sequencing.

### MiRNA selection

MiRNAs predicted to target *TARDBP*, *FUS/TLS* and *RGNEF* 3’UTRs were selected using miRanda software. Further, the sequence of the miRNA had to be perfectly complementary to the miRNA recognition element (MRE) from +2 to +7. Novel miRNAs currently not found with the miRanda program were manually checked to see if their seed sequence had a MRE within the 3’UTR of *TARDBP*, *FUS/TLS* and *RGNEF*. We only considered those miRNAs for which we identified MREs within the 3’UTR isoforms of *TARDBP*, *FUS/TLS* and *RGNEF* within in the spinal cord tissue.

### Real-time PCR

Total miRNA extractions were performed on ventral lumbar spinal cord tissue using the mirVana miRNA extraction kit according to manufactures instructions (Life Technologies Inc., Ambion, Carlsbad, CA, USA). Yield and purity of the miRNA extracts were measured using spectrophotometry (Nanodrop, ThermoFisher Scientific, Burlington, ON, Canada), while integrity was measured using Bioanalyzer (Aligent Technologies Canada Inc., Missasauga, ON, Canada) analysis. MiRNA extracts were reversed transcribed and then subjected to real-time PCR using miRCURY LNA™ Universal RT microRNA PCR (Exiqon, Woburn, MA, USA) and ExiLENT SYBR Green master mix (Exiqon, Woburn, MA, USA) kits, respectively, according to manufacturer’s instructions. To detect novel miRNAs, miRNAs extracts went under reverse transcription using the Taqman microRNA reverse transcriptase kit (Life Technologies Inc., Applied Biosystems, Forest City, CA, USA), and then were pre-amplified using the Taqman PreAmp Master Mix Kit (Life Technologies Inc., Applied Biosystems, Forest City, CA, USA) followed by real-time PCR with the TaqMan Universal PCR Master Mix (×2) no AmpErase UNG (Life Technologies Inc., Applied Biosystems, Roche, Branchburg, NJ, USA). The 7900 HT Real Time PCR system was used to read PCR outputs. Relative expression of miRNAs was normalized to an internal control (miR-16-5p), followed by comparison of the relative expression of candidate miRNAs between ALS cases and a control population using the 2^-ΔΔCT^ method. Negative values show down-regulation and positive values up-regulation of the expression. Statistical significance was determined using Student’s t-test, and samples were considered significantly different if *p*  < 0.05.

### Fluorescent in situ hybridization (FISH)

Neuropathologically normal human lumbar spinal cord tissue was formalin-fixed paraffin-embedded and cut into 7 μm sections. Samples were UV treated overnight prior to the experiment to reduce lipofuscin-induced autofluorescent signaling. FISH of miRNAs was performed as described before [25]. Probes for miRNA detection were designed with double DIG labels (Exiqon, Woburn, MA, USA), and were targeted by a DIG-HRP secondary antibody (1:100; Roche, Indianapolis, IN, USA) and Tyramide Signal Amplification tagged with a Cy3 fluorophore (PerkinElmer, Waltham, MA, USA). Olympus FV1000 confocal microscope was used to observe miRNA expression within spinal motor neurons.

### Cell culture and plasmid construction

HEK293T and SH-SY5Y cells were cultured in Dulbecco’s Modified Eagle’s Media (DMEM) containing 10% Fetal Bovine Serum (FBS). Cells were incubated at 37 °C with 5% CO_2_.

3’UTR isoforms of RNA-binding proteins identified in the human spinal cord tissue were individually cloned into the pmirGLO vector in between SalI and NheI restriction enzyme sites and downstream from the firefly luciferase gene (Promega, Madison, WI, USA). Site-directed mutagenesis assays were done by adding a two-nucleotide mutation within the +2 and +3 positions of each miR-194 or miR-b2122 MRE using the Site-Directed Mutagenesis Kit II (Aligent Technologies Canada Inc., Missasauga, ON, Canada) according to the manufacturer’s instructions. Primers used are showed in Additional file 1: Table S1. Mutations were carefully designed to ensure no changes to mRNA secondary structure using RNAfold WebServer (http://rna.tbi.univie.ac.at/cgi-bin/RNAfold.cgi).

### Luciferase assay

HEK293T cells were seeded into 96 well plates (9000 cells per well) 24 h prior to transfection. Cells were co-transfected with 3.5 fmol of pmirGLO plasmid and 100 nM of miRNA mimics according to the Lipofectamine 2000 protocol (Life Technologies Inc., Invitrogen, Burlington, ON, Canada). Luciferase activity was measured 24 h post-transfection using the Dual-GLO Luciferase Assay System (Promega, Madison, WI, USA). Firefly activity was normalized to renilla activity. Experimental design and normalization of data was performed as previously described [26]. Data was quantified as relative difference from the control, and expressed as mean ± SEM. Statistical significance was determined by performing Student’s t-test, and was considered significantly different if *p* < 0.05.

### Relative quantitative RT-PCR

To determine the effects of miR-194 and miR-b2122 on the luciferase mRNA expression when it contained the 3’UTR of *TARDBP*, *FUS/TLS* or *RGNEF*, HEK293T cells were seeded into 24 well plates (20,000 cells per well) 48 h prior to transfection. Cells were co-transfected with 20.6 fmol of pmirGLO plasmid and 100 nM of miRNA mimics according to the Lipofectamine 2000 protocol (Life Technologies Inc., Invitrogen, Burlington, ON, Canada). 24 h after transfection, total RNA extraction was performed using TRIzol reagent (Life Technologies Inc., Ambion, Carlsbad, CA, USA) followed by first-strand cDNA synthesis (Life Technologies Inc., Invitrogen, Burlington, ON, Canada) and PCR amplification of firefly and renilla cDNA as previously described [13]. Data was quantified as relative difference from the control, and expressed as mean ± SEM. Statistical significance was determined by performing Student’s t-test, and was considered significantly different if *p* < 0.05.

To identify whether miR-194 and miR-b2122 could regulate the endogenous mRNA expression of these three RNA-binding proteins within a neuronal-derived cell line, SH-SY5Y cells were seeded into 6-well plates (500,000 cells per well) 24 h prior to the transfection. 100 nM of miRNA mimics and anti-miRs were then either transfected individually or co-transfected. 24 h after transfection total RNA extraction was performed using TRIzol reagent (Life Technologies Inc., Ambion, Carlsbad, CA, USA) followed by cDNA synthesis (Life Technologies Inc., Invitrogen, Burlington, ON, Canada). Quantitative PCR (qPCR) to determine the relative change in endogenous mRNA expression of *TARDBP*, *FUS/TLS* and *RGNEF* was performed using following primers: TARDBP *for*: 5′-CAG GGT GGG TTT GGT AAC GT-3′ *rev*: 5′-AAA GCC CCC ATT AAA ACC AC-3′; FUS/TLS *for*: 5′-TCG GGA CCA AGG ATC ACG TC-3′ *rev*: 5′-ATC TGG TTT AGG GGC CTT ACA CTG-3′; RGNEF *for*: 5′-AGG AAC GCA ATA ACT GGA TGA GAC G-3′ *rev*: 5′-TTC CAC CTT CTC CCC TGC ATC AG-3′; 18S RNA *for*: 5′-AGT TGG TGG AGC GAT TTG TC-3′ *rev*: 5′-TTC CTC GTT CAT GGG GAA TA-3′. All expression profiles were normalized to 18S RNA levels prior to comparison. One-way ANOVA followed by a Tukey’s post-hoc was used to determine statistical differences in endogenous mRNA expression, and samples were significantly different if *p* < 0.05.

### Western blot analysis

SH-SY5Y cells were seeded into 6-well plates (500,000 cells per well) 24 h prior to the experiment. 100 nM of miRNA mimics and inhibitors were then either transfected individually or co-transfected. 48 h after transfection total protein extraction was performed using NP40 lysis buffer containing proteinase inhibitors (cOmplete, Roche, Indianapolis, IN, USA), followed by sonication. Samples were suspended in loading buffer and proteins were denatured at 90 °C for 5 min. Samples were run on a 12% SDS-gel, and transferred to a nitrocellulose membrane. To measure endogenous levels of TDP-43, FUS/TLS and RGNEF, the membrane was probed with either anti-TDP-43 (1:2500; Proteintech, 10,782–2-AP), anti-FUS/TLS (1:3000; Proteintech, 11,570–1-AP), or anti-RGNEF (1:1000; Abcam, ab157095) rabbit antibodies, respectively. Blots were then probed with a HRP-secondary antibody (goat anti-rabbit; 1:5000; Life Technologies Inc., Invitrogen, Burlington, ON, Canada). Blots were stripped using stripping buffer (2% SDS, 62.5 mM Tris-HCl, 100 mM β-mercaptoethanol, pH 6.8) and re-probed for GAPDH using anti-GAPDH rabbit antibody (1:2500; Abcam, ab9485). Relative protein expression of TDP-43, FUS/TLS and RGNEF were normalized to GAPDH expression levels. One-way ANOVA followed by a Tukey’s post-hoc was used to determine statistical differences in endogenous protein expression, and samples were significantly different if *p* < 0.05.

## Results

### A small group of miRNAs contain MREs within the mRNA 3’UTR of *TARDBP*, *FUS/TLS,* and *RGNEF*

**Fig. 1:**
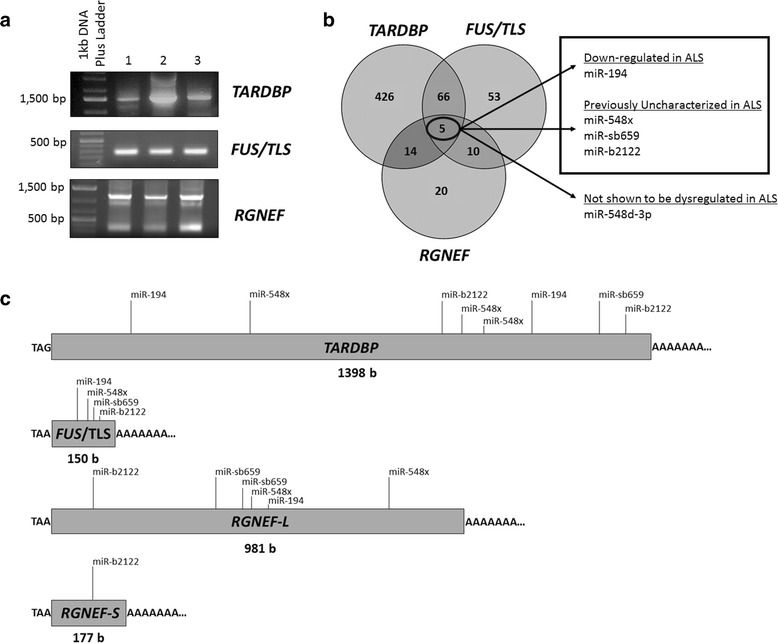
A small group of miRNAs have MREs within the 3’UTRs of *TARDBP*, *RGNEF* and *FUS/TLS*. **a** 3’RACE PCR identified the 3’UTRs of *TARDBP, FUS/TLS* and *RGNEF* that are expressed in the human spinal cord tissue within three different control samples. One isoform of both *TARDBP* and *FUS/TLS* were identified with a length of 1398 and 150 bases, respectively. Two isoforms of *RGNEF,* which we have termed *RGNEF*-short (*RGNEF*-S) and *RGNEF*-long (*RGNEF*-L) 3’UTRs, were identified in all three subjects running at 177 and 981 bases, respectively. Bands in figures appear higher than actual 3’UTR size, as primers were designed upstream from stop codon. **b** Five miRNAs were identified to have binding sites within the *TARDBP*, *FUS/TLS* and *RGNEF* mRNA 3’UTRs. MiR-548d-3p in previous work has shown to have no dysregulation in sALS, and thus, four miRNAs (outlined in the black box) went under further study. **c** Schematic of all *TARDBP*, *FUS/TLS* and *RGNEF* 3’UTRs identified within human spinal cord, and location of MREs for miRNA candidates

Spinal cord tissue from neurologically intact individuals was used to determine the 3’UTR isoform(s) of *TARDBP*, *FUS/TLS* and *RGNEF* being expressed. *TARDBP* consistently showed one 3’UTR isoform across all human samples with a length of 1398 bp (BC095435.1) (Fig. 1a) [27]. Subject two appeared to have a higher band, but we were unable to confirm a longer 3’UTR through sequencing, and for that reason, we focused only on the transcript that was consistently expressed across all samples.

*FUS/TLS* contained one 3’UTR isoform within three different control cases, which was 150 bp in length (Fig. 1a) (NM_004960). While *RGNEF* appeared to have three 3’UTR isoforms in each control case, we were only able to confirm the top and bottom bands through sequencing. The two *RGNEF* 3’UTR isoforms that were confirmed had a length of 177 bp and 981 bp, which we termed *RGNEF*-short and *RGNEF*-long, respectively (Fig. 1a). Both short and long 3’UTRs of *RGNEF* have been previously described (NM_001244364.1 and NM_0010804079.2, respectively).

Subsequently, we identified 5 miRNAs which had MREs within the 3’UTR of all three of these RNA-binding proteins. However, our previous work has indicated that miR-548d-3p was not dysregulated in sALS cases, and thus was eliminated from further analysis. We also previously observed that miR-194 is down-regulated in sALS patients [13], while miR-b2122, miR-sb659 and miR-548× have not been analyzed for dysregulation within sALS patients (Fig. 1b, c). The latter four miRNAs were thus of interest for further analysis.

### MiR-194 and miR-b2122 are down-regulated in the spinal cord tissue of sALS patients

**Fig. 2:**
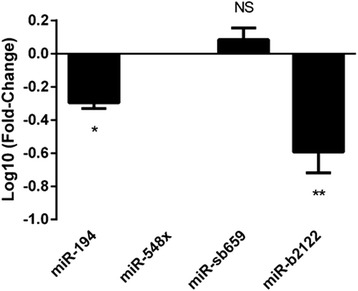
Differential expression of candidate miRNAs within the spinal cord of sALS patients. Candidate miRNA expression was examined in ventral spinal cord tissue of sALS patients (*n* = 8) and control subjects (*n* = 5). MiR-194 and miR-b2122 were significantly down-regulated in sALS patients, while miR-sb659 showed no difference and miR-548× was not expressed in the spinal cord tissue. Data was expressed as Log10 (fold-change) ± SEM, and significance was determined using Students t-test (** = *p* < 0.01, *p < 0.05, NS = *p* > 0.05)

**Fig. 3:**
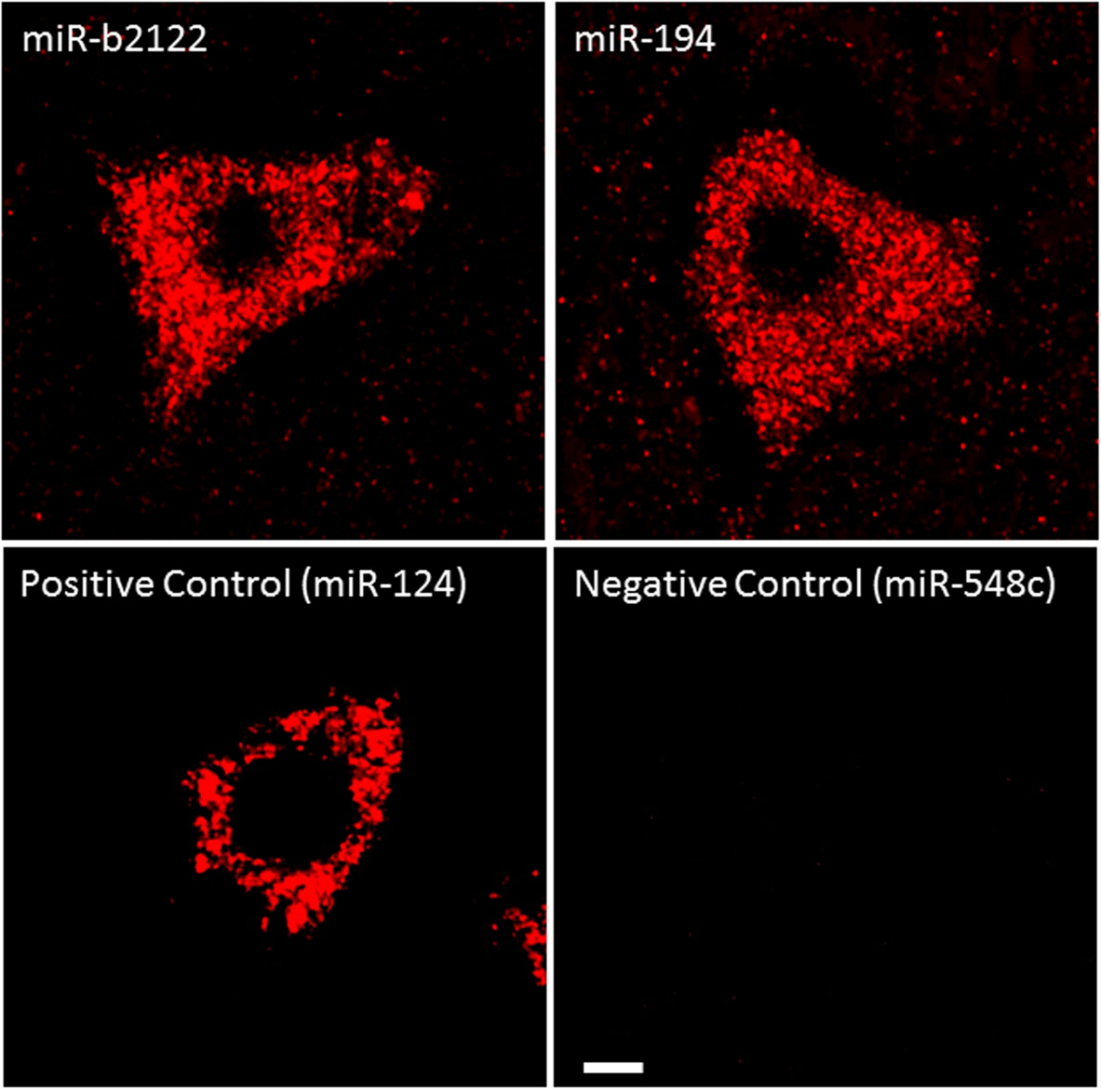
MiR-194 and miR-b2122 are expressed in human spinal motor neurons. Ventral human spinal cord of control tissue was analyzed using FISH to determine the expression of miR-194 and miR-b2122 within motor neurons. Both miRNAs showed strong positive staining within motor neurons. MiR-124 and miR-548c were used as positive and negative controls, respectively. Scale bar represents 10 μm

We characterized the relative expression of miR-194, miR-548×, miR-sb659 and miR-b2122 within the spinal cord tissue of sALS patients compared to control subjects. Using real-time PCR, we observed that miR-194 and miR-b2122 were significantly down-regulated in sALS patients (Fig. 2). The down-regulation of miR-194 is consistent with what we reported previously using TaqMan Array [13]. Using FISH, we confirmed that both miR-194 and miR-b2122 were strongly expressed within human spinal motor neurons of control samples with little to no non-motor neuronal expression, suggesting that the down-regulation of these two miRNAs is likely motor neuron specific (Fig. 3).

### MiR-b2122 regulates a reporter linked to either *TARDBP*, *FUS/TLS*, or *RGNEF* 3’UTR

**Fig. 4:**
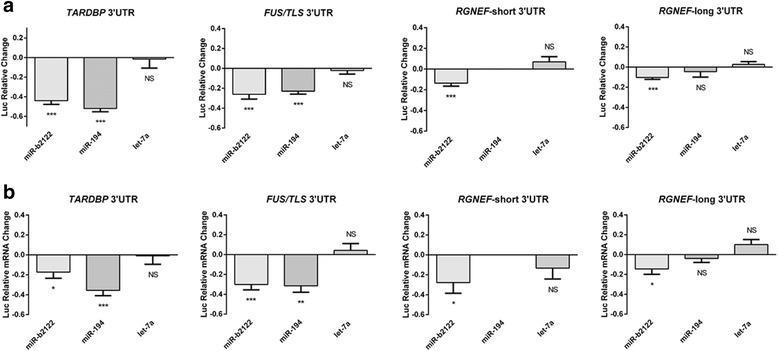
MiR-b2122 reduces firefly luciferase activity when it contains either the *TARDBP*, *FUS/TLS* or *RGNEF* 3’UTR. HEK293T cells were transfected with a pmirGLO plasmid containing the 3’UTR of one of the RNA-binding proteins of interest either with or without miR-194 or miR-b2122. PmirGLO plasmid without any 3’UTRs were also transfected with or without miRNAs of interest to determine the miRNAs effect on the plasmid itself. **a** Reporter gene assay revealed miR-b2122 reduced firefly activity when it contained either the *TARDBP*, *FUS/TLS*, or *RGNEF*-short/long 3’UTR, whereas miR-194 down-regulated firefly levels when it contained either *TARDBP*, *FUS/TLS*, but not *RGNEF*-long. MiR-194 has no MRE in RGNEF-short, and thus, the interaction between the two was not examined. **b** RT-qPCR results showed similar suppression of mRNA levels as seen to the luciferase activity observed in the reporter gene assay. Let-7a was used as a negative control for these experiments. Firefly was normalized to renilla luciferase, and then further normalized to account for the effect of each miRNA on the pmirGLO vector to determine the exact effect that each miRNA has on each 3’UTR. Each miRNA was compared to its own individual control based on the normalization of the data. Data is expressed as sample mean ± SEM, and significance was determined using a Student’s t-test (*** = *p* < 0.001, ** = *p* < 0.01, * = *p* < 0.05, NS = *p* > 0.05)

A reporter gene assay was used to examine the effect of miR-194 and miR-b2122 on the regulation of firefly luciferase protein when it contained the 3’UTR of either *TARDBP*, *FUS/TLS*, or *RGNEF* that we identified within the human spinal cord. MiR-b2122 significantly reduced firefly protein activity when it contained either the *TARDBP*, *FUS/TLS*, *RGNEF-*short, or *RGNEF*-long 3’UTR, whereas miR-194 down-regulated firefly protein activity only when it contained either the *TARDBP* or *FUS/TLS* 3’UTR, and had no effect when it contained the *RGNEF-*long 3’UTR (Fig. 4a). MiR-194 did not contain an MRE within the *RGNEF*-short 3’UTR and thus the interaction between these two components was not examined. Further, to determine if miR-194 and miR-b2122 could also alter luciferase mRNA levels, we performed RT-PCR analysis. The results in the RT-PCR analysis matched the down-regulation seen by these two mRNAs in the luciferase reporter gene assay, indicating that the effect of these miRNAs involves regulation of the levels of mRNA species (Fig. 4b).

**Fig. 5:**
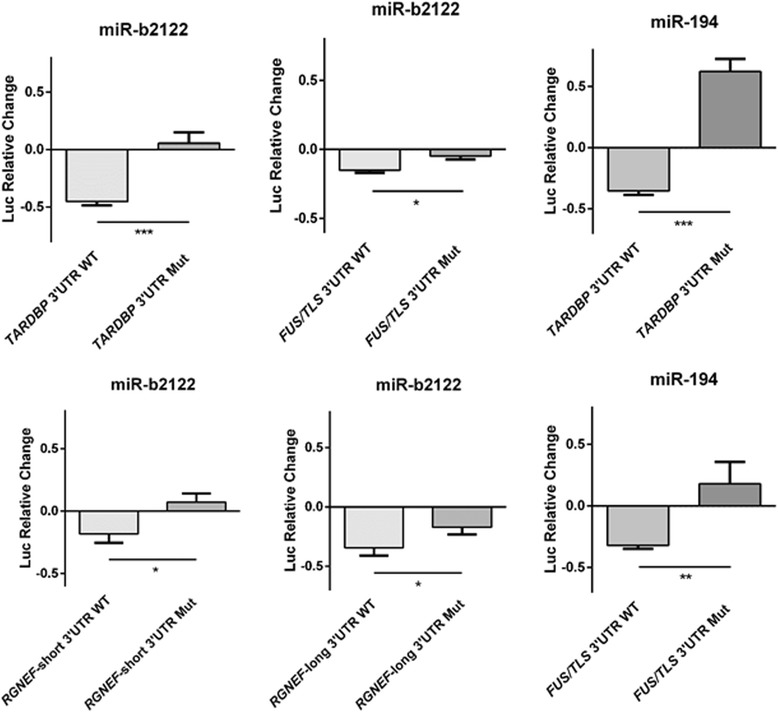
MiR-b2122 and miR-194 directly interact with their 3’UTR targets. HEK293T cells were co-transfected with either the pmirGLO plasmid containing the wild-type 3’UTR, or the 3’UTR mutant, and either with or without the miRNA of interest. Mutations within the MRE of miR-b2122 in the *TARDBP*, *FUS/TLS*, and *RGNEF* 3’UTRs, and the MRE of miR-194 in the *TARDBP* and *FUS/TLS* 3’UTRs abolished each miRNAs ability to reduce firefly activity. Firefly was normalized to renilla, and then further normalized to the effect of each miRNA on the pmirGLO vector to determine the miRNAs exact effect on the 3’UTR. Data is expressed as sample mean ± SEM, and significance was determined using a student’s t-test (*** = *p* < 0.001, ** = *p* < 0.01, * = *p* < 0.05)

To study if miR-194 and miR-b2122 were regulating firefly luciferase by directly interacting with the 3’UTR, we mutated two nucleotides within the MRE sites of miR-194 and miR-b2122. Mutating the miR-b2122 MRE sites within either the *TARDBP*, *FUS/TLS*, *RGNEF*-short, or *RGNEF*-long 3’UTR significantly abolished the ability of miR-b2122 to reduce firefly luciferase activity. Similarly, mutating the miR-194 MRE sites within either *TARDBP* or *FUS/TLS* ablated miR-194 down-regulation of the firefly protein (Fig. 5). These findings indicate that both miR-194 and miR-b2122 directly interact with their 3’UTR targets to regulate gene expression.

### MiR-b2122 regulates endogenous TDP-43, FUS/TLS and RGNEF within a human neuronal cell line

Next, we decided to determine if miR-b2122 and miR-194 regulate the endogenous mRNA expression of *TARDBP, FUS/TLS* and *RGNEF* within a human neuronal-derived cell line – SH-SY5Y cells. SH-SY5Y cells express the *TARDBP*, *FUS/TLS* and *RGNEF* 3’UTR isoforms we identified with in the spinal cord. SH-SY5Y cells showed multiple bands in the *TARDBP* lane, but we were not able to confirm the top two bands through sequencing, only the 1398 b 3’UTR isoform identified in spinal cord, which also appears to be dominantly expressed in SH-SY5Y cells (Additional file 2: Figure S1a). Also, endogenous expression of miR-b2122 and miR-194 in SH-SY5Y cells was confirmed through real-time PCR (Additional file 2: Figure S1b).

**Fig. 6:**
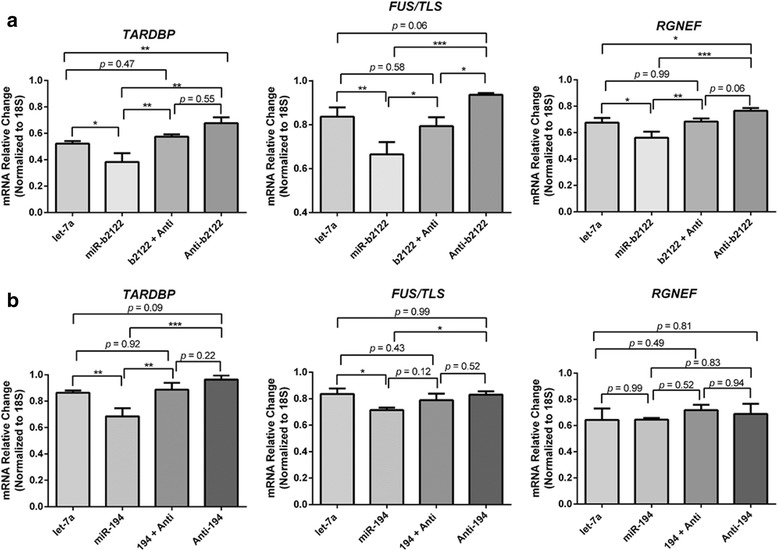
MiR-b2122 regulates mRNA expression of *TARDBP*, *FUS/TLS* and *RGNEF* in a human neuronal cell line. SH-SY5Y cells were transfected individually with miR-194 or miR-b2122, or co-transfected with miR-194 or miR-b2122, and their anti-miRs. Transfection of let-7a was used as a negative control. **a** Transfection of miR-b2122 significantly down-regulates the mRNA levels of *TARDBP*, *FUS/TLS* and *RGNEF*, while co-transfection of miR-b2122 with its anti-miR led to a recovery in the mRNA levels. Transfection of the anti-miR alone lead to increased mRNA expression of all three RNA-binding proteins (**b**) Transfection of miR-194 alone resulted in reduction of *TARDBP* and *FUS/TLS*, which was abolished when it was co-transfected with its anti-miR. No effect was observed on RGNEF when miR-194 and/or its anti-miR were transfected. Data was expressed as the mean ± SEM, and significance was determined using a one-way ANOVA followed by a Tukey’s post-hoc. (*** = *p* < 0.001, ** = *p* < 0.01 * = *p* < 0.05)

Transfection of miR-b2122 lead to a significant down-regulation in *TARBDP*, *FUS/TLS* and *RGNEF* mRNA levels. Further, co-transfection of miR-b2122 with its anti-miR abrogates the down-regulation of these transcripts via miR-b2122. Transfection of the anti-miR of miR-b2122 alone lead to an up-regulation in mRNA levels of all three genes (Fig. 6a). The up-regulation observed with the addition of the anti-miR, suggests that miR-b2122 does regulate these RNA-binding proteins endogenously within this neuronal cell line. Let-7a was used as negative control, as we showed it has no effect on the endogenous mRNA and proteins levels of these three genes (Additional file 3: Figure S2).

Further, we also transfected miR-194 and/or its anti-miR within SH-SY5Y cells. Similar to the reporter gene assays, miR-194 only reduced *TARDBP* and *FUS/TLS* endogenous mRNA levels with no effect on *RGNEF* mRNA levels, which was abolished when co-transfecting miR-194 with its anti-miR (Fig. 6b). Also, only adding the anti-miR of miR-194 caused a strong trend towards up-regulation of *TARDBP* mRNA expression, but no change in the *FUS/TLS* transcript levels, suggesting miR-194 might play a role in regulating *TARDBP* gene expression within SH-SY5Y cells. Overall, these results suggest that miR-b2122 is the central regulator of *TARDBP*, *FUS/TLS* and *RGNEF* mRNA expression.

**Fig. 7:**
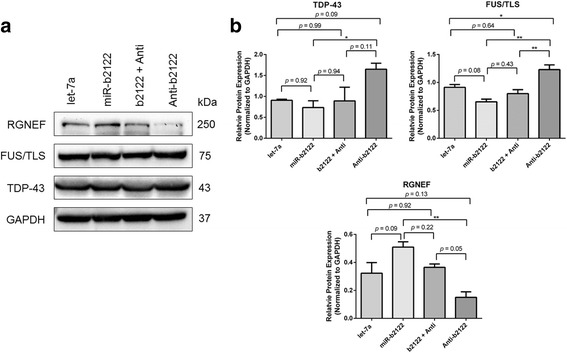
MiR-b2122 alters protein levels of TDP-43, FUS/TLS and RGNEF within a human neuronal cell line. Changes in protein levels of TDP-43, FUS/TLS and RGNEF were studied when SH-SY5Y cells were transfected with either miR-b2122, miR-b2122 plus its anti-miR, or the anti-miR alone. Transfection of let-7a was used as a negative control. **a** Western blot showing expression of TDP-43, FUS/TLS, RGNEF, and GAPDH (**b**) Quantification of Western blots for TDP-43, FUS/TLS and RGNEF protein levels. TDP-43 and FUS/TLS show small reductions in protein levels when transfected with miR-b2122 alone, while their protein levels increased when the anti-miR is added. Differences in protein levels when miR-b2122 or the anti-miR are added alone are significantly different for both TDP-43 and FUS/TLS. RGNEF has increased and decreased protein levels when either miR-b2122 or its anti-miR are added alone, respectively, and these differences are significantly different from one another. Protein levels were normalized to GAPDH. Data was expressed as the mean ± SEM, and significance was determined using a one-way ANOVA followed by a Tukey’s post-hoc (** = *p* < 0.01, * = *p* < 0.05)

To determine whether the alteration in mRNA levels was associated with alterations in protein expression, we examined protein levels of TDP-43, FUS/TLS and RGNEF post-transfection of miR-b2122 (Fig. 7a). MiR-b2122 alone had no significant effect on the protein levels of TDP-43 within the cell, but when the anti-miR alone was added, there was a strong trend towards up-regulation of TDP-43. This up-regulation was significantly different from when miR-b2122 was transfected alone, suggesting that endogenous miR-b2122 is likely participating keeping TDP-43 protein at steady-state levels, but loss of this miRNA leads to increase TDP-43 protein output (Fig. 7b). Transfection of miR-b2122 alone showed a strong trend towards the down-regulation in FUS/TLS protein levels, which was abrogated when the anti-miR was co-transfected with miR-b2122. The transfection of the anti-miR of miR-b2122 alone did lead to a significant up-regulation of FUS/TLS protein levels, indicating miR-b2122 regulates protein synthesis of FUS/TLS endogenously (Fig. 7b).

Interestingly, miR-b2122 had the reverse effect on the protein levels of RGNEF as compared to the changes observed at the mRNA level (Fig. 7b). Transfection of miR-b2122 alone lead to increased RGNEF protein levels, which was reduced to the control levels when miR-b2122 was co-transfected with its anti-miR. Transfection of the anti-miR alone lead to reduced levels of RGNEF compared with let-7a. While these results were not significantly different from the negative control, there was a significant difference in RGNEF protein levels between when either miR-b2122 or the anti-miR were transfected alone. All data was compared to let-7a (negative control), as we showed it, has no effect on the proteins levels of these three genes (Additional file 4: Figure S3). Overall, these results indicate that miR-b2122 can regulate FUS/TLS protein expression, while having minor changes on TDP-43 and RGNEF protein levels.

### ALS mutation in *FUS/TLS* 3’UTR is located in miR-b2122 MRE

**Fig. 8:**
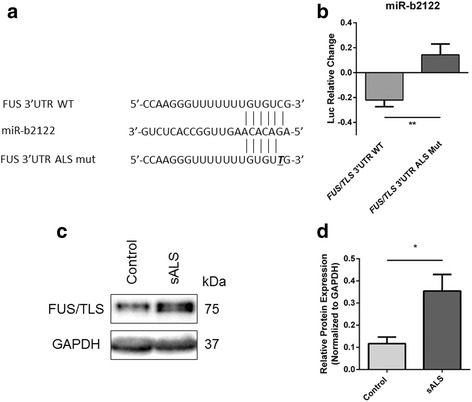
ALS-associated mutation within *FUS/TLS* 3’UTR inhibits the ability for miR-b2122 to reduce firefly activity. HEK293T cells were co-transfected with either the pmirGLO plasmid containing the wild-type *FUS/TLS* 3’UTR, or the mutated form, and either with or without miR-b2122. **a** ALS-associated mutation (*c.108 C > T) affects the +2 binding site of the miR-b2122 MRE. **b** ALS-associated mutation within the *FUS/TLS* 3’UTR inhibits miR-b2122 from reducing firefly activity. Firefly expression was normalized to renilla expression, and then further normalized to account for the effect miR-b2122 on the pmirGLO vector itself to determine the miRNAs exact effect on the 3’UTR. **c** Western blot of FUS/TLS protein expression in the spinal cord of sALS cases versus control subjects. **d** Quantification of western blot. Data is expressed as sample mean ± SEM, and significance was determined using a Student’s t-test (** = *p* < 0.01)

Previously, mutations within the *FUS/TLS* 3’UTR were found within ALS patients, all of which lead to the overexpression and increased cytoplasmic mislocalization of FUS/TLS protein [20]. Interestingly, one of these mutations (*c.108C > T) is located in the +2 position of the MRE for miR-b2122 (Fig. 8a), suggesting this would critically affect the ability of miR-b2122 to bind and reduce *FUS/TLS* expression. We sought to investigate whether this mutation would affect the ability to regulate firefly expression when the firefly gene was linked to the *FUS/TLS* 3UTR that contained the *c.108C > T mutation. Indeed, this mutation significantly abolished the ability for miR-b2122 to reduce the firefly expression, compared to when the firefly gene contained the wild-type *FUS/TLS* 3UTR (Fig. 8b). This result implies that FUS/TLS would be overexpressed without proper regulation of miR-b2122 via direct interaction with the 3 UTR.

Based on the previous result, we decided to examine if sALS cases that showed a down-regulation in miR-b2122 have an increase in FUS/TLS expression. In sALS cases that showed a down-regulation of miR-b2122 there was a 3-fold increase in FUS/TLS protein expression (Fig. 8c, d), suggesting a relationship between reduced levels of miR-b2122 and increase FUS/TLS expression.

## Discussion

In this study, we identified miR-b2122 to be a central regulator of ALS-linked RNA-binding proteins TDP-43, FUS/TLS and RGNEF. We showed that miR-b2122 was significantly down-regulated within the spinal cord tissue of sALS patients, and specifically expressed within motor neurons. MiR-194, which was also found be down-regulated in sALS patients, regulates the mRNA expression of *TARDBP* and *FUS/TLS*, but not *RGNEF*. Together, our data introduces a novel miRNA (miR-b2122) to sALS pathology, and indicates that the down-regulation of this miRNA in sALS could affect a regulatory network of RNA-binding proteins within motor neurons, contributing to the disease pathology.

The 3’UTRs for *TARDBP*, *FUS/TLS*, and *RGNEF* identified in spinal cord match those that have been previously described; however, for *TARDBP* we were only able to describe one 3’UTR isoform, while previous authors have described multiple. The *TARDBP* 3’UTR isoform we described matches the pA1 transcript isoform [27]. Whether this is the only isoform expressed in spinal cord, or a limitation of our technique, the pA1 isoform is known to be the dominant transcript expressed in steady-state conditions, and has been shown to be the main isoform for TDP-43 protein synthesis [28–30]. Further, it has been hypothesized that the pA1 isoform is the one overexpressed in ALS [28], providing another reason why we decided to focus on the pA1 isoform, and its interactions with miR-194 and miR-b2122.

MiR-194 is a well-known tumor suppressor, and reduced levels of miR-194 has been linked to both cancer and diabetes [31–36]. Interestingly, the dysfunctional pathways identified within both of these diseases relate to those described in ALS [33, 36]. For example, miR-194 expression has been shown to be switched off by NF-kB - a proinflammatory transcription factor that has been associated with ALS progression via increase activation in astrocytes and microglia [33, 37, 38]. Further, overexpression of TDP-43 has been related to an increase in NF-kB activation [38]. In this study, reduction of miR-194 leads to increased levels of *TARDBP* mRNA, and thus, through its regulation of TDP-43, miR-194 may be part of an inflammatory regulatory network that contributes to ALS progression.

Since miR-b2122 was a novel miRNA identified by our group previously [39], this is the first pathway in which this miRNA has been implicated. Our data suggests that the down-regulation of miR-b2122 would lead to a significant increase in *TARDBP*, *FUS/TLS* and *RGNEF* mRNA levels in sALS patients. This is consistent with the increase of *TARDBP* mRNA and protein levels observed in sALS patients [38]. Further, rodent models overexpressing wild-type human FUS/TLS and TDP-43 do develop age-related motor deficiencies and cytoplasmic protein aggregation in motor neurons similar to that seen in ALS cases [40–43]. However, the latter models look at the overexpression of a single gene, when it is the dysregulation of both expression and localization of multiple RNA-binding proteins which contributes to the disease progression. This makes miR-b2122 an intriguing miRNA, as its down-regulation in sALS would contribute to the overexpression and dysregulation of multiple RNA-binding proteins involved in its pathogenesis.

While TDP-43, FUS/TLS and RGNEF protein levels showed discrete changes when compared to the negative control, there were significant changes between when miR-b2122 and its anti-miR were transfected alone, suggesting that either overexpression, or reduced activity of miR-b2122, does in fact alter protein levels. These noticeable changes to the TDP-43, FUS/TLS and RGNEF protein levels when miR-b2122 levels are increased or decreased, suggests that chronic changes to miR-b2122 activity might have more drastic effects on protein levels within the cell over-time.

Interestingly, RGNEF protein levels went in the opposite direction of the mRNA levels within our study. While rare to see inverse correlations between mRNA and protein levels of a single gene, it is not unprecedented [44, 45]. This could imply when miR-b2122 binds to the *RGNEF* 3’UTR, its role is to maintain low levels of mRNA while keeping the transcript in a translationally stable state, and thus, loss of its binding stabilizes the mRNA molecule, but leaves the transcript in a translationally silent state. The latter phenomenon is a common one seen within stress and transport granules within neurons [46–48]. However, this would suggest that there is competition between miR-b2122 and another miRNA, or RNA-binding protein at the *RGNEF* 3’UTR which would need further investigation.

We sought to determine whether an ALS mutation located in the MRE of miR-b2122 within the *FUS/TLS* 3’UTR effected the ability of miR-b2122 to reduce gene expression. Clinically, the patient identified with this FUS/TLS 3’UTR mutation (*c.108C > T) had limb onset ALS with severe limb weakness and respiratory difficulties. Previously, fibroblast cells cultured from the ALS patient with this mutation showed an overexpression of FUS/TLS mRNA and protein, and an increase in cytoplasmic localization – two factors believed to contribute to ALS development [20]. Despite identifying these phenotypes there was no clear mechanism to why this may happen. In this study, we showed that loss of direct interaction between miR-b2122 and the *FUS/TLS* 3’UTR may play a critical role in FUS/TLS overexpression. Further, we were able to show that reduced levels of miR-b2122 in sALS spinal cord seems to be related to an increase in FUS/TLS protein expression. In addition, reduction of the levels of miR-b2122 in a neuronal cell line (SH-SY5Y) using anti-b2122 hindered the ability for miR-b2122 to reduce endogenous *FUS/TLS* leading to an overall increase in both mRNA and protein levels.

In this study, we have not only provided an explanation of the significance for reduced levels of miR-b2122 in sALS, but provide a molecular link showing the importance of the interaction between miR-b2122 and the *FUS/TLS* 3’UTR. Thus, dysregulation of miR-b2122 either through reduce levels or mutations within the MRE could be a major contributing factor to FUS/TLS dysregulation and pathogenesis in ALS. In a different study, a group examined another ALS-related mutation within the *FUS/TLS* 3’UTR, which lead to an overexpression of FUS/TLS. This aberrant expression of FUS/TLS was attributed to the loss of its interaction with miR-141/200a due to the 3’UTR mutation [21]. These findings emphasize the importance of examining mutations outside of the coding regions, as alterations within the 3’UTR can have drastic effects on both protein expression and localization [49, 50].

While it is interesting to note the relationship between the dysregulation of RNA-binding proteins and miRNAs, it is still unclear how miRNAs, like miR-b2122 and miR-194, become reduced in sALS. However, there is strong evidence suggesting that the miRNA biogenesis pathway is disrupted in sALS, as both TDP-43 and FUS are crucial parts of miRNA production [18, 19]. More specifically, it appears that the dysregulation in miRNA biogenesis happens at the level of DICER, as it is the mature miRNA form, and not the pre-miRNA form, showing a global down-regulation in sALS [15]. Also, ALS-linked mutations in TDP-43 and FUS affect miRNA biogenesis specifically at the level DICER [15]. Thus, it is plausible that TDP-43 and/or FUS could regulate the biogenesis of miR-b2122 and miR-194, suggesting that a negative feedback loop between RNA-binding proteins and miRNAs exists, and that it is the loss of this negative feedback loop that drives, at least in part, sALS disease progression.

## Conclusions

It has been previously shown that the pathogenesis of sALS likely does not rely on the dysregulation of a single RNA-binding protein, but a combination of TDP-43, FUS/TLS and RGNEF, as they co-aggregate with each other in motor neurons of sALS patients [6]. In the current study, we have identified a single miRNA that regulates all three of these RNA-binding proteins. The observation that miR-b2122 is down-regulated in sALS suggests that this miRNA may play an essential role in the pathogenic mechanism of sALS. As we further look at those miRNAs related to ALS, we start developing an understanding of a miRNA network critical for motor neuron function, which we have termed MotomiRs [16]. Further, it would be intriguing to know whether these miRNAs play a role in closely related neurodegenerative diseases, including primary lateral sclerosis (PLS), spinal muscular atrophy (SMA), or frontotemporal dementia (FTD). Based on the current study, miR-b2122 should be added to the already established list of MotomiRs, as it regulates a network of RNA-binding proteins essential for motor neuron function, and its regulation could potentially contribute to motor neuron degeneration in ALS.

## Additional files

Additional file 1: Table S1. Site-directed mutagenesis primers for *TARDBP*, *FUS/TLS* and *RGNEF* 3’UTRs. (DOCX 16 kb)

Additional file 2: Figure S1. 3’UTR isoforms of RNA-binding proteins, and miR-194 and miR-b2122 are expressed in SH-SY5Y cells. (A) 3’RACE PCR showing TARDBP, FUS/TLS and RGNEF 3’UTR isoforms expressed in SH-SY5Y cells. FUS/TLS and RGNEF isoforms match those expressed in human spinal cord. TARDBP showed multiple isoforms, but only the 1398b isoform identified in spinal cord could be confirmed by sequencing. (B) Real-time PCR indicating the expression of miR-194 and miR-b2122 in SH-SY5Y cells. (TIFF 171 kb)

Additional file 3: Figure S2. Let-7a has no effect on mRNA levels of TARDBP, FUS/TLS, or RGNEF within SH-SY5Y cells. Let-7a was transfected into SH-SY5Y cells to determine if it changed the basal mRNA levels of TARDBP, FUS/TLS or RGNEF, and was compared to a non-transfected control. The data indicated no significant change in the transcript levels of either TARDBP (p=0.64), FUS/TLS (*p*=0.51), or RGNEF (*p*=0.74) between the two conditions. Data is expressed as sample mean ± SEM, and significance was determined using a Student’s t-test.(TIFF 160 kb)

Additional file 4: Figure S3. Let-7a has no effect on protein levels of TDP-43, FUS/TLS, or RGNEF within SH-SY5Y cells. Let-7a was transfected into SH-SY5Y cells to determine if it changed the basal protein levels of TDP-43, FUS/TLS or RGNEF, and was compared to a non-transfected control. The data indicated no significant change in the protein levels of either TDP-43 (*p*=0.71), FUS/TLS (*p*=0.28), or RGNEF (*p*=0.87) between the two conditions. Data is expressed as sample mean ± SEM, and significance was determined using a Student’s t-test. (TIFF 215 kb)

## Abbreviations

ALS: Amyotrophic lateral sclerosis
fALS: Familial ALS
FISH: Fluorescent in situ hybridization
FUS/TLS: Fused in sarcoma/translocation in liposarcoma
MRE: miRNA Recognition element
RACE: Rapid amplification of cDNA ends
RGNEF: Rho guanine nucleotide exchange factor
sALS: Sporadic ALS
TARDBP: TAR DNA-binding protein gene
TDP-43: TAR DNA-binding protein, 43 kDa
UTR: Untranslated region
