# Supplementary material for: Novel miR-b2122 regulates several ALS-related RNA-binding proteins
Source: Mol Brain. 2017 Oct 2;10:46. doi: 10.1186/s13041-017-0326-7 (PMC5625648; doi:10.1186/s13041-017-0326-7)
Supplement: Supplementary file 1 — Site-directed mutagenesis primers for TARDBP, FUS/TLS and RGNEF 3’UTRs. (DOCX 16 kb) [file 13041_2017_326_MOESM1_ESM.docx]

Table S1. Site-directed mutagenesis primers for *TARDBP*, *FUS/TLS* and *RGNEF* 3’UTRs.

| 3’UTR | MRE of interest | Site (bp from stop codon) | Primers |
| --- | --- | --- | --- |
| *TARDBP* | miR-194 | 200 | *for***:** 5’-GGA ATT TTA TAA GTT TTG TT***G T***AT GAA AG GTT GAA ATA TTG-3’  *rev*: 5’-CAA TAT TTC AAC CTT TCA T***AC*** AAC AAA ACT TAT AAA ATT CC-3’ |
|  |  | 1145 | *for*: 5’-GGT GTG TGT TCT CTT CTG TT***G T***TG ATA TGT AAG TGT GGC AAT G  *rev*: 5’-CAT TGC CAC ACT TAC ATA TCA ***AC***A ACA GAA GAG AAC ACA CAC C-3’ |
|  | miR-b2122 | 906 | *for*: 5’-CTT GGC GAG ATG TG***A T***TC TCA ATC CTG TGG-3’  *rev*: 5’-CCA CAG GAT TGA GA***A T***CA CAT CTC GCC AAG-3’ |
|  |  | 1330 | *for*: 5’-GTT TGC TGC AGT TCT GTG ***AT***C TGT GCT TGG ATG C-3’  *rev*: 5’GCA TCC AAG CAC AG***A T***CA CAG AAC TGC AGC AAA C-3’ |
| *FUS/TLS* | miR-194 | 57 | *for*: 5’-GTC CTG TAC CCA GTG TT***C A***CC TCG TTA TTT TGT AAC-3’  *rev*: 5’-GTT ACA AAA TAA CGA GG***T G***AA CAC TGG GTA CAG GAC-3’ |
|  | miR-b2122 | 111 | *for:* 5’-CCC AAG GGT TTT TTT GTG ***AG***G GAC TAT GTA ATT GTA AC-3’  *rev*: 5’GTT ACA ATT ACA TAG TCC ***CT***C ACA AAA AAA CCC TTG GG-3’ |
| *RGNEF-*short | miR-b2122 | 96 | *for*: 5’-GTG TGA TTG TCT GTG ***GA***C AAA TTG CTT TAA G-3’  *rev*: 5’-CTT AAA GCA ATT TG***T C***CA CAG ACA ATC ACA C-3’ |
| *RGNEF*-long | miR-b2122 | 96 | *for*: 5’-GTG TGA TTG TCT GTG ***AA***C AAA TTG CTT TAA G-3’  *rev*: 5’-CTT AAA GCA ATT TG***T T***CA CAG ACA ATC ACA C-3 |
| *FUS/TLS* ALS mt | miR-b2122 | 111 | *for*: 5’-CCC AAG GGT TTT TTT GTG T***T***G GAC TAT GTA ATT GTA AC-3’  *rev*: 5’-GTT ACA ATT ACA TAG TCC ***A***AC ACA AAA AAA CCC TTG GG-3’ |

Underline areas within the primer sequence represent the MRE, while bolded/italicized nucleotides represent mutation made.

**Figure S1. 3’UTR isoforms of RNA-binding proteins, and miR-194 and miR-b2122 are expressed in SH-SY5Y cells.** (A) 3’RACE PCR showing *TARDBP*, *FUS/TLS* and *RGNEF* 3’UTR isoforms expressed in SH-SY5Y cells. *FUS/TLS* and *RGNEF* isoforms match those expressed in human spinal cord. *TARDBP* showed multiple isoforms, but only the 1398b isoform identified in spinal cord could be confirmed by sequencing. (B) Real-time PCR indicating the expression of miR-194 and miR-b2122 in SH-SY5Y cells.

**Figure S2. Let-7a has no effect on mRNA levels of *TARDBP*, *FUS/TLS*, or *RGNEF* within SH-SY5Y cells.** Let-7a was transfected into SH-SY5Y cells to determine if it changed the basal mRNA levels of *TARDBP*, *FUS/TLS* or *RGNEF*, and was compared to a non-transfected control. The data indicated no significant change in the transcript levels of either *TARDBP* (p=0.64), *FUS/TLS* (p=0.51), or *RGNEF* (p=0.74) between the two conditions. Data is expressed as sample mean ± SEM, and significance was determined using a Student’s t-test.

**Figure S3. Let-7a has no effect on protein levels of TDP-43, FUS/TLS, or RGNEF within SH-SY5Y cells.** Let-7a was transfected into SH-SY5Y cells to determine if it changed the basal protein levels of TDP-43, FUS/TLS or RGNEF, and was compared to a non-transfected control. The data indicated no significant change in the protein levels of either TDP-43 (p=0.71), FUS/TLS (p=0.28), or RGNEF (p=0.87) between the two conditions. Data is expressed as sample mean ± SEM, and significance was determined using a Student’s t-test.
