# Supplementary figures and images for: Novel miR-b2122 regulates several ALS-related RNA-binding proteins
Source: Mol Brain. 2017 Oct 2;10:46. doi: 10.1186/s13041-017-0326-7 (PMC5625648; doi:10.1186/s13041-017-0326-7)

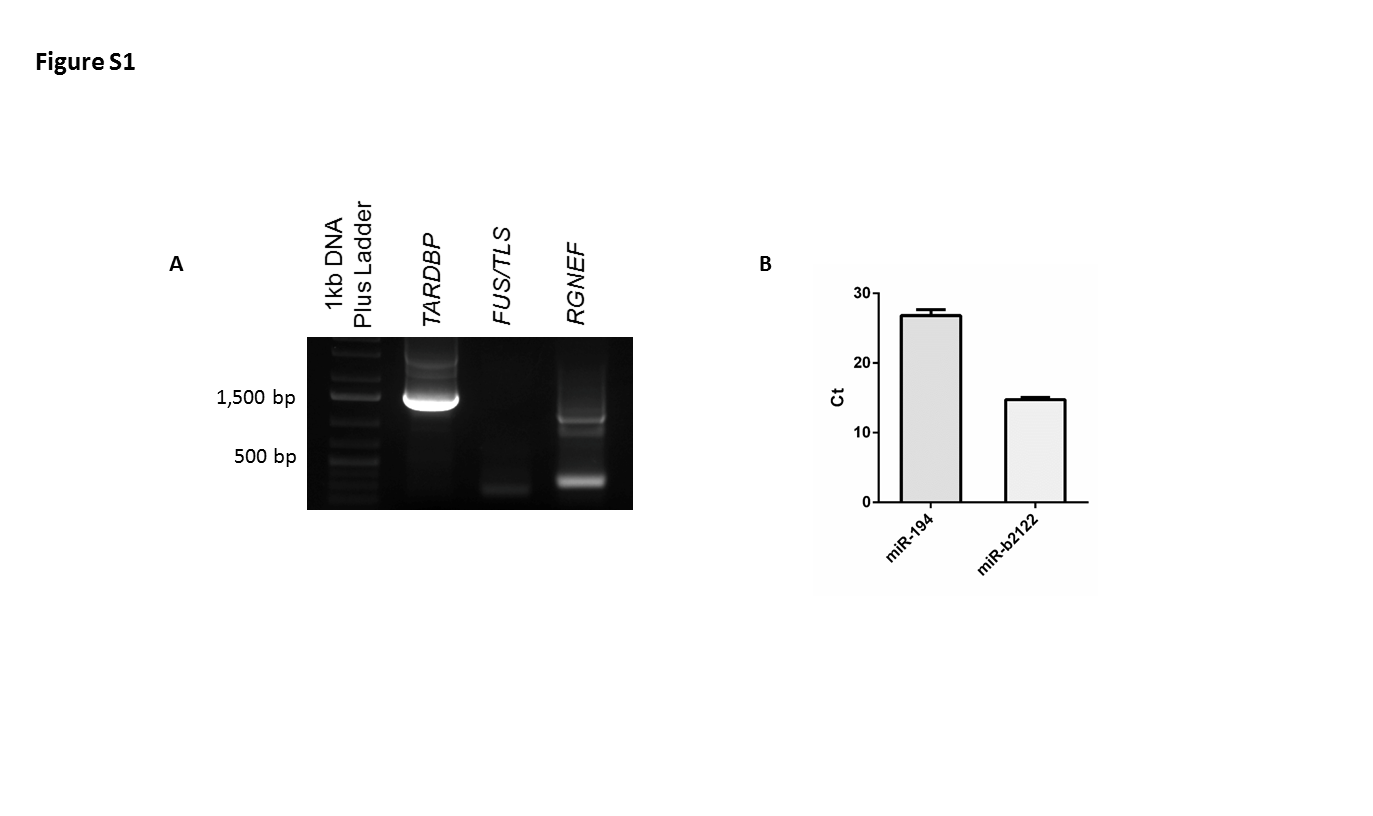

Supplement: Supplementary file 2 — 3’UTR isoforms of RNA-binding proteins, and miR-194 and miR-b2122 are expressed in SH-SY5Y cells. (A) 3’RACE PCR showing TARDBP, FUS/TLS and RGNEF 3’UTR isoforms expressed in SH-SY5Y cells. FUS/TLS and RGNEF isoforms match those expressed in human spinal cord. TARDBP showed multiple isoforms, but only the 1398b isoform identified in spinal cord could be confirmed by sequencing. (B) Real-time PCR indicating the expression of miR-194 and miR-b2122 in SH-SY5Y cells. (TIFF 171 kb) [file 13041_2017_326_MOESM2_ESM.tif]

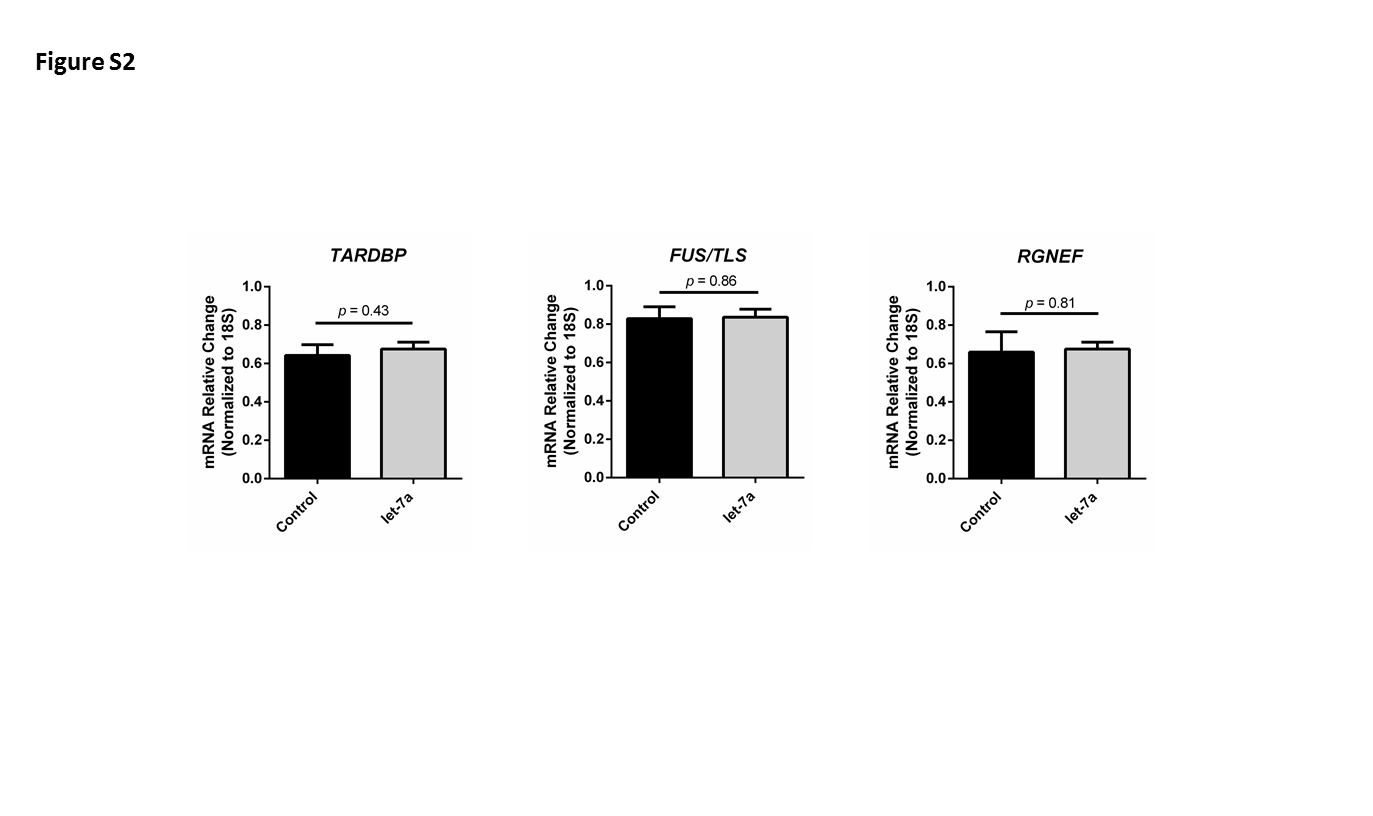

Supplement: Supplementary file 3 — Let-7a has no effect on mRNA levels of TARDBP, FUS/TLS, or RGNEF within SH-SY5Y cells. Let-7a was transfected into SH-SY5Y cells to determine if it changed the basal mRNA levels of TARDBP, FUS/TLS or RGNEF, and was compared to a non-transfected control. The data indicated no significant change in the transcript levels of either TARDBP (p=0.64), FUS/TLS (p=0.51), or RGNEF (p=0.74) between the two conditions. Data is expressed as sample mean ± SEM, and significance was determined using a Student’s t-test.(TIFF 160 kb) [file 13041_2017_326_MOESM3_ESM.tif]

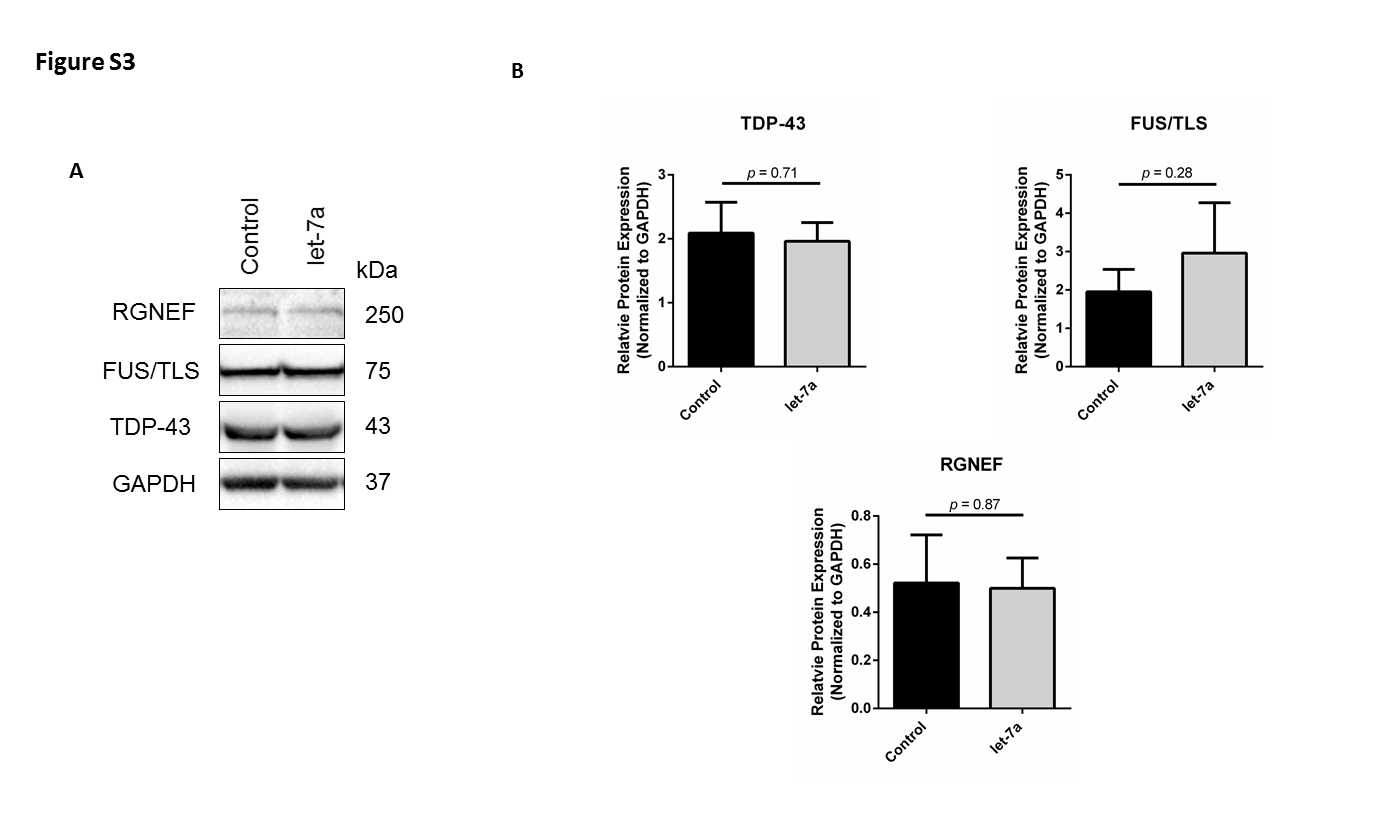

Supplement: Supplementary file 4 — Let-7a has no effect on protein levels of TDP-43, FUS/TLS, or RGNEF within SH-SY5Y cells. Let-7a was transfected into SH-SY5Y cells to determine if it changed the basal protein levels of TDP-43, FUS/TLS or RGNEF, and was compared to a non-transfected control. The data indicated no significant change in the protein levels of either TDP-43 (p=0.71), FUS/TLS (p=0.28), or RGNEF (p=0.87) between the two conditions. Data is expressed as sample mean ± SEM, and significance was determined using a Student’s t-test. (TIFF 215 kb) [file 13041_2017_326_MOESM4_ESM.tif]
